# Dynamic Multidimensional Analysis of Active Aging in Italy: Effects of Regional Economic Growth

**DOI:** 10.1093/geroni/igaf122.2330

**Published:** 2025-12-31

**Authors:** Davide Lucantoni, Valerio Intraligi, Bernard Steinman, Mirko Di Rosa, Giovanni Lamura, Francesco Barbabella, Marco Socci, Andrea Principi

**Affiliations:** INRCA - National Institute on Health and Science of Aging, Ancona, Marche, Italy; INRCA - National Institute on Health and Science of Aging, Ancona, Marche, Italy; University of Wyoming, Laramie, Wyoming, United States; INRCA - National Institute on Health and Science of Aging, Ancona, Marche, Italy; INRCA - National Institute on Health and Science of Aging, Ancona, Marche, Italy; INRCA - National Institute on Health and Science of Aging, Ancona, Marche, Italy; INRCA - National Institute on Health and Science of Aging, Ancona, Marche, Italy; INRCA - National Institute on Health and Science of Aging, Ancona, Marche, Italy

## Abstract

The United Nations and European policy bodies encourage national governments to promote and implement active aging (AA), to address social and economic challenges linked to population aging. A crucial feature of AA as a construct is its multidimensionality. Indeed, a measuring tool of AA is the Active Ageing Index (AAI), a composite index which encompasses four domains (i.e., 1. Employment; 2. participation in society; 3. independent healthy and secure living; and 4. capacity and enabling environment for AA) for a total of 22 indicators within those domains. The aim of this study was to assess whether and to what extent AA was implemented in a multidimensional way within all the (20) Italian regional governments, over the period 2007-2018. An Italian ad-hoc version of the AAI was used. First, it was measured the percentage contribution of each AA domain to the overall growth of AA during the study’s time-frame. Then, ordinary least squares regression models were employed, to assess whether regional economic growth was correlated with the observed changes in the different AAI domains. Results show that changes in the AAI scores in Italy’s administrative regions are predominantly driven by changes in the employment domain. Thus, the implementation of multidimensionality was mostly neglected. Moreover, regional economic growth resulted to be positively associated with changes in the employment domain and negatively associated with changes in the participation in society domain. These results entail important implications for policy-making, as they highlight the need to promote AA multidimensionality more extensively.

